# Points on the Repeal of the Compulsory Vaccination Law of the State of New York*Read before the Anti-Compulsory Vaccination League.

**Published:** 1895-05

**Authors:** B. Fincke

**Affiliations:** Brooklyn, N. Y.


### POINTS ON THE REPEAL OF THE COMPULSORY VACCINATION LAW OF THE STATE OF NEW YORK.*

B. Fincke, M. D., Brooklyn, N. Y.

1. The law giving to the Boards of Health—*i. e.*, to the Commissioners—arbitrary power to order vaccination of everybody whenever they should see fit, is a veritable force bill, depriving any inhabitant of the United States of his right to his own body and soul by compulsory vaccination. Article VI Amendments to the Constitution insures to every person life, liberty, or property, which no State shall deprive him of without due process of law, and the equal protection of the law within its jurisdiction.

Far too much power is given to the Commissioners by that odious law, and the official for our city has proved twice his capacity to interpret it, not in the sense of equity and discretion as implied by the term "may" in the law, but in the sense of tyranny and arbitrariness which is abhorrent to our system of

---

* Read before the Anti-Compulsory Vaccination League.

government (Gaynor). For, in the last year, according to the official report, the whole number of small-pox cases from January to July 1st amounted to 385 cases, and this was the dreadful epidemic in a city of nearly a million inhabitants, against which the Commissioner opposed a veritable epidemic of vaccination, creating at least 400,000 cases of allopathic poisoning, in sending out his cohorts on a campaign of raiding the people in the dead of night, assisted by a strong police force, which committed such deeds of violence in the sacred homes of the people as never have been perpetrated in the worst old monarchical countries of Europe.

According to the report, every ward of Brooklyn has been invaded by at least two cases from January, 1892, to February, 1894. This remark seems to imply that small-pox is a chronically epidemic disease of Brooklyn, justifying continued compulsory vaccination.

To these chronic cases come three acute cases in the Eastern District, just discovered when the Supreme Court in General Term reversed the admirable decision of Judge Gaynor, denying the right of compulsory vaccination. The next evening 25 vaccinists, backed by 75 policemen, were sent out again to vaccinate everybody in the infected district, even the passers-by in the streets, whether previous vaccinated or not. Surely all these cases, even the 385 from January to July, last year, did not justify the declaration of an epidemic in this large city. Or will the Commissioner claim, that after having vaccinated 400,000, to have saved all the rest of 600,000 from small-pox!

2. Vaccination is made the condition of public education. An ugly discrimination is practically made by this compulsion. For it excludes all those from the public schools who can pay for private tuition of their children if they prefer them not to be vaccinated. This is all wrong. The rich and the poor ought to sit side by side in the public school. But it comes especially hard upon the workingmen who can ill afford to pay school-money while they are taxed all the same for public education.

What a farce is made of this true republican and democratic measure, that every child should go to the public school to be educated to be a good citizen of the republic!

3. This despotic law elevates the old allopathic school of medicine to a State institution, against which there is no help if it is not repealed. The idea of compulsory vaccination in and out of schools is essentially an allopathic measure incompatible with homœopathic practice. The homœopathic school finds that vaccination is against sound principles of medicine and corrupting the health of the whole population by poisoning them with something which has no bearing at all upon small-pox, much less for protection from it. If a doubt arises in criminal cases, the court gives to the criminal the benefit of the doubt. The criminal according to that odious law is not the Commissioner who is empowered thereby to suppress the people if so he is disposed, but the people who rise against the oppression. As the contest about the value of the tyrannical measure is going on, let the people at least have the benefit of the doubt and exonerate it from the compulsion in and out of school by repealing the law.

4. The assumed value of vaccination by the allopathic profession of which the Commissioner is an offspring, has nothing to do with this enforcement of compulsory vaccination. This trick which is calculated to obtain succor from the panic created by declaring a disease epidemic is contemptible and should be resisted by all right-minded men. It is absurd for the inhabitant of the ward where the Commissioner is located (in the Western district) to feel assured of not becoming infected when 400,000 persons of the Eastern district are vaccinated under the most outrageous circumstances that could be imagined. If there is such a bliss in vaccination why does the Commissioner not raid the Brooklyn Heights or The Hill? Why does he not send his minions just as well into the City Hall and the municipal buildings around it? Why does he not arrest every one walking in the street in the pursuit of his business?

5. The idea by universal vaccination to stop and stamp out the small-pox disease is childish. The child wants to reach the moon with its little hands. The susceptibility of a person for small-pox is not under the control of the Commissioner, who like the profession to which he belongs, does not acknowledge the fundamental principles of healing, viz., the homœopathic, or he would use homœopathic means for protection. The infection of small-pox does not proceed by means of microbes, bacteria or bacilli, but in an insensible, unassignable manner which escapes the efforts of the bacteriologist and pathologist. The poverty of allopathic treatment is nowhere more conspicuous than in the treatment of small-pox, for we have it on the authority of the Pathological Society of Brooklyn, which indorsed the Commissioner, that the only scientific treatment of small-pox is vaccination and re-vaccination and continually repeated re-vaccination. No medical treatment is given in the small-pox hospitals for small-pox. The windows are open the weather may be as it will. This comes from patients who have been there.

Nay, vaccination as inoculation has become the Alpha and Omega and the prevailing type of general allopathic practice. They have not physiology enough to know that the sick can be reached by safer and gentler means than by hypodermic injection of poisonous substances.

6. If vaccination and re-vaccination is the only treatment for small-pox patients and the mainstay of prevention, then the whole matter is in a nut-shell: the immense power of a Commissioner of Health is used for incalculable injury to the public, and should be curtailed by repealing that obnoxious law. If this is offensive in the nostrils of the reigning schools, make the most of it! In such things pertaining to life, liberty, and happiness of the people, guaranteed by the Declaration of Independence and the Constitution, we must obey God more than man, and persist upon the repeal of compulsory vaccination in and out of our public schools.

